# Advances in Drug Delivery Nanosystems Using Graphene-Based Materials and Carbon Nanotubes

**DOI:** 10.3390/ma14051059

**Published:** 2021-02-24

**Authors:** Josef Jampilek, Katarina Kralova

**Affiliations:** 1 Institute of Neuroimmunology, Slovak Academy of Sciences, Dubravska Cesta 9, 845 10 Bratislava, Slovakia; 2 Regional Centre of Advanced Technologies and Materials, Czech Advanced Technology and Research Institute, Palacky University, Slechtitelu 27, 783 71 Olomouc, Czech Republic; 3 Institute of Chemistry, Faculty of Natural Sciences, Comenius University, Ilkovicova 6, 842 15 Bratislava, Slovakia; kata.kralova@gmail.com

**Keywords:** drugs, carbon nanotubes, drug delivery nanosystems, graphene, graphene oxide, graphene quantum dots, nanoparticles

## Abstract

Carbon is one of the most abundant elements on Earth. In addition to the well-known crystallographic modifications such as graphite and diamond, other allotropic carbon modifications such as graphene-based nanomaterials and carbon nanotubes have recently come to the fore. These carbon nanomaterials can be designed to help deliver or target drugs more efficiently and to innovate therapeutic approaches, especially for cancer treatment, but also for the development of new diagnostic agents for malignancies and are expected to help combine molecular imaging for diagnosis with therapies. This paper summarizes the latest designed drug delivery nanosystems based on graphene, graphene quantum dots, graphene oxide, reduced graphene oxide and carbon nanotubes, mainly for anticancer therapy.

## 1. Introduction

Each active pharmaceutical ingredient (API, drug substance) is formulated for administration to prevent, treat, or diagnose into a dosage form that corresponds to the desired method of use. Dosage forms can be divided according to their physical state (solid, semi-solid, liquid dosage forms and transdermal patches) or according to the route of administration (gastrointestinal, parenteral and topical). The dosage form thus enables/facilitates the manufacture, preparation, storage (increases stability) and administration of drugs and their properties and can favorably influence, for example, the disintegration of the tablet in the body, the overall bioavailability, the slow release of the API, etc. [1,2,3,4]. It is possible to state that three generations of dosage forms are distinguished. The 1st generation represents the majority of current drugs on the market. It is characteristic of them that the profile of plasma concentrations over time is influenced only by pharmacokinetic processes (absorption, distribution, metabolism, elimination) and the physicochemical properties of API (solid state form, solubility). The formulation itself releases all the drugs contained in it very quickly. The 2nd generation of formulations is characterized by controlled/sustained-release, i.e., in addition to pharmacokinetic processes; the profile of plasma concentrations of the API achieved is also influenced by the properties of the formulation. The main advantage of this is the ability to release the drug slowly at a constant rate, which allows stable plasma concentrations to be maintained for some time. These formulations may be further modified to release the initial (shock) dose immediately after administration. The task of the 3rd generation dosage forms with targeted distribution is to introduce the active substance molecule by the shortest route into the target tissue to the receptors. The API does not come into contact with tissues where it could cause side/toxic effects (e.g., preparations used to treat cancer) [2,3,5].

**Figure 1:**
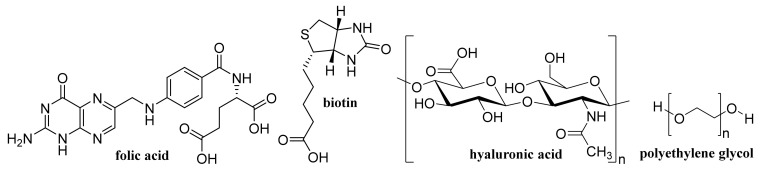
Examples of compounds used to modify the surface of nanosystems.

The use of higher generation drug delivery systems improves the efficacy of many existing drugs and allows the introduction of new therapies. Efforts to miniaturize them from macro-dimensions (>1 mm) to micro-, submicro- to nano-dimensions can be traced back to the 1990s, with great progress in recent years being made with the massive introduction of nanotechnologies [5,6,7]. Extremely popular are various nanoemulsions of lipidoid formations or colloidal nanodispersions of nanocrystals, i.e., nanoliposomes, solid lipid nanoparticles (NPs), and various other nanovesicles, dendrimers, polymer systems, tubules and quantum dots (QDs) are used as drug carriers [8,9,10,11,12,13,14,15,16,17,18]. Currently, nanoforms made from non-toxic biodegradable materials are preferred; however, in the case of nanoformulations for cancer therapy or diagnosis, inorganic carriers such as NPs of metals, metal oxides, metalloids and carbon are also used, which often potentiate the effect of the API itself [19,20,21,22,23,24,25,26,27,28,29,30]. It is important to mention that drugs in the nanoform acquire unique physicochemical properties and in particular their bioavailability after oral administration is modified, thanks to improved permeability through membranes [6,31,32,33,34,35,36,37,38,39,40]. In addition, drug delivery nanosystems (nanoDDSs) make it easy to achieve a targeted distribution, whether it is a passive distribution based on the particle size of the NPs or the so-called EPR (Enhanced Permeability and Retention factor) effect, active distribution, i.e., by modifying the NP surface with an antibody, ligand, etc., or in the case of magnetic NPs by an external magnetic field. The surface of nanoDDSs for active targeting can be modified with monoclonal antibodies or fragments thereof, short peptides, oligonucleotides, lectins, etc. Very often, albumin, aptamer A10, hyaluronic acid (HA, Figure 1), folic acid (FA, Figure 1) or biotin (Figure 1) are used. Total hydrophilicity and “invisibility” against phagocytes are ensured by surface modification using polyethylene glycol (PEG, Figure 1) by so-called PEGylation [12,37,41,42,43,44,45,46,47,48,49,50].

**Figure 2:**
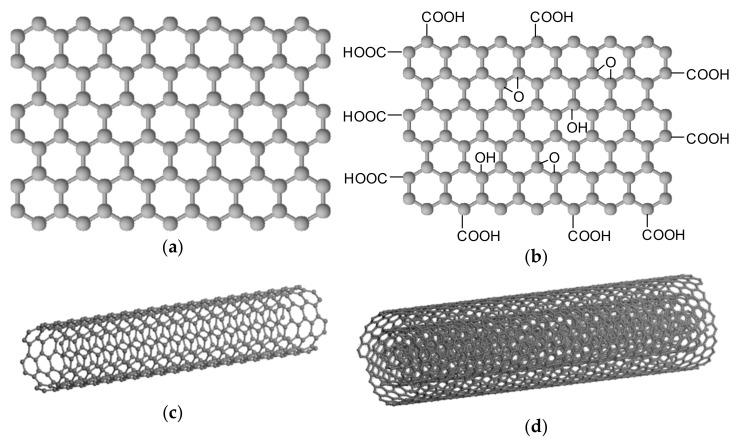
Structures of graphene (**a**) and graphene oxide (**b**), single-walled carbon nanotubes (**c**) and multi-walled carbon nanotubes (**d**).

Carbon is one of the most abundant elements on Earth. In nature, it occurs mainly as part of a huge amount of organic matter and in the form of carbon dioxide can be found in the atmosphere. Pure carbon occurs in nature in several crystallographic modifications such as graphite, diamond, lonsdaleite and chaoite. For example, fullerene C_60_ or the non-crystalline mineraloid Shungite can also be found in nature. Other allotropic modifications of carbon include graphene (and its various oxidized or reduced forms including graphene QDs), graphyne, graphdiyne, and carbon nanotubes [51,52,53,54]. Graphene (GR, Figure 2a) is formed by six-membered cycles arranged in planar carbon layers (sp^2^ hybridization). In 2010, the Nobel Prize in Physics was awarded for the discovery of a two-dimensional GR material. The surface of graphene is hydrophobic. Graphene nanosheets are composed of more than 10 graphene sheets below 100 nm in thickness. GR has very remarkable properties, which makes GR a very promising material for a variety of bioapplications [26,55,56,57,58]. Graphene oxide (GO, Figure 2b) is prepared by oxidation of GR, i.e., by introducing carbonyl, hydroxyl and epoxide groups on planar surfaces and edges of GR carbon plates. GO can be prepared by various methods [26,59,60,61,62,63]. Subsequently, GO can be reduced by various methods, thus reducing the number of oxygen groups, to produce reduced graphene oxide (rGO), which will be similar in properties to the pattern GR [26,64,65]. Graphyne was theoretically predicted by Baughman in 1987. These are carbon structures in the hybridization of sp^1^ and sp^2^. Graphyne forms monoatomic layers [66]. Graphdiyne has a similar structure to graphyne, which is supplemented by diacetylene bridges [67]. Carbon nanotubes (CNTs) are carbon allotropes with a cylindrical structure with open or closed ends. They can be classified based on the number of concentric layers of rolled graphene sheets: single-walled CNTs (SWCNTs, Figure 2c) generally showing an outer diameter of 0.8–2 nm, and multiwalled CNTs (MWCNTs, Figure 2d) with outer diameters of 5–20 nm. Their lengths can range from 100 nm to several centimeters [68]. Other characteristics of GR, GO, rGO and CNTs can be found, e.g., [26,69,70,71,72].

Graphene-based nanomaterials and CNTs are designed to help deliver or target drugs more efficiently. They are being investigated for therapeutic applications, especially for the treatment of cancer, but also for the development of new diagnostics and nanosensors and are expected to help combine molecular imaging for diagnosis with therapy, especially in the development of treatment strategies in oncology. An important area of interest for these materials is their toxicity, and therefore ways are being sought to reduce the toxicity of these materials so that they can be used for biomedical applications. This is helped by various surface modifications, strict particle size ranges, etc. [26,73,74,75,76,77,78,79,80,81,82,83,84,85,86,87,88,89,90,91,92,93,94,95].

Another form of carbon can be considered activated carbon (AC, charcoal) that is composed primarily of aromatic configurations of carbon atoms joined by random cross-linkages—its sheets or groups of atoms stacked unevenly in a disorganized manner. It is characterized by a high porosity and a high-surface area reflected in the good adsorptive ability of this material. The spaces between the AC crystallites constitute the microporous structure with a large internal surface area, usually of 250–2500 m^2^/g [96]. The pores represent approximately 5% of the total surface area of the AC; their volume in the activated carbon ranges generally between 0.2 and 0.5 cm^3^/g and their surface area from 0.5 to 2 m^2^/g [96,97]. Charcoal is mainly used as a material for the production of various sorbents applied as part of filters, for example in the decontamination of air, water and soil. As one of the few forms of carbon, it has a traditional use in medicine, where it is again highly valued for its high sorption properties, not only as an oral adsorption detoxifier, but it is also currently used for the production of a new generation of so-called sorption dressings. Nanocharcoal has also been prepared in recent years and finds the same uses as activated carbon [98,99,100,101,102,103].

Considerable efforts have been made in the last decade to explore the biomedical use of graphene-based nanomaterials and carbon nanotubes, especially in the intelligent administration of anticancer drugs [26,104,105]. Unfortunately, regulatory authorities have not approved a single product for launching to the market where these carbon-based nanomaterials are used. Statistics show that liposomes have the highest proportion in medical nanoproducts (>33%), followed by nanocrystals (23%), emulsions (14%), polymer-iron complexes (9%) and micelles (6%), while carbon-based nanomaterials have a very small share [106,107,108,109]. One of the main reasons for this, compared to successfully approved nanoDDS-based drugs, is the fact that, in general, carbon-based nanomaterials are considered to be one of the most dangerous nanomaterials with a high potential to penetrate cell walls due to their physicochemical properties, size and shape (e.g., risks of inflammatory reactions, pulmonary fibrosis and DNA damage) [74,110,111,112,113]. On the other hand, the functionalization of these nanomaterials leads to the suppression of toxic effects, which enables the successful integration of these nanomaterials into the biomedical field [26,104,105,114,115]. The aim of this paper is therefore to summarize the latest designed graphene-based (i.e., based on graphene, graphene quantum dots, graphene oxide, reduced graphene oxide) and carbon nanotube drug delivery systems mainly for anticancer therapy.

## 2. Graphene Quantum Dots

Zero-dimensional graphene quantum dots (GQDs) consisting of a single to a few layers of GR sheets with lateral dimensions of <10 nm, which are characterized by superb photostability, tunable fluorescence due to their remarkable quantum confinement and edge effects, and water solubility, being also non-toxic and biocompatible, can be successfully fabricated using top-down strategies including chemical exfoliation, electrochemical exfoliation, hydrothermal/solvothermal exfoliation and microwave/ultrasound-assisted exfoliation, or using bottom-up strategies including carbonization/pyrolysis, stepwise organic synthesis/cage opening and chemical vapor deposition (in detail see in [116,117]). GQDs can be applied in various bioimaging applications, including fluorescence and two-photon fluorescence imagining, magnetic resonance imaging (MRI), and dual-modal imaging [79,117,118,119,120] and they are also suitable to be used as photoluminescence, electrochemiluminescence or electrochemical sensors and for the sensing of key neurotransmitters (dopamine, tyrosine, epinephrine, norepinephrine, serotonin and acetylcholine) [121,122]. GQDs, due their ability to cross the blood-brain barrier (BBB) and biocompatibility, could be considered superb delivery systems for loaded drugs through the bloodstream, across the BBB and up to the brain and can be successfully used in neuroscience diagnostics and therapeutics such as photothermal and photodynamic therapy alone or in combination with chemotherapy [16,119,123,124]. Drug delivery-release modes of GQDs-based drug delivery systems including enhanced permeability and retention, (EPR)-pH delivery-release, ligand-pH delivery-release, EPR-photothermal delivery-release, and core/shell-photothermal/magnetic thermal delivery-release modes were overviewed by Jha et al. [125], Levy et al. [126] and Zhao et al. [127].

### 2.1. Unmodified GQDs

Although a non-cytotoxic dose of 15 μg/mL of GQDs (50 nm) did not cause a considerable reduction in the viability of MCF-7, HUVEC, and KMBC/71 cells 4 and 24 h post exposure, the GQDs greatly altered the expression level of genes involved in breast tumor development and metastasis (miR-21, miR-29a, Box, Bcl2 and PTEN) in the cells as well as mitochondrial activity at the cellular level, suggesting that altered cell fate and susceptibility may result in deviation in the desired outcome of GQDs application [128]. In complexes of *N*,*N*-dimethylphenylenediamine-derivatized nitrilotriacetic acid vanadyl compounds loaded on GQDs, the vanadyl compounds were packed closely on one side of the GQD sheets, possibly through the π–π stacking mechanism. These complexes showed in vitro membrane permeability comparable with that of GQDs and were less toxic than GQDs. In vivo experiments performed on type 2 diabetic mice showed that the complexes of vanadyl compounds with GQD showed a delayed glucose-lowering profile and after three-week treatment exhibited a more significant impact on insulin enhancement and β-cell protection than the vanadyl compound alone [129].

### 2.2. Capped/Encapsulated/Coated GQDs

**Figure 3:**
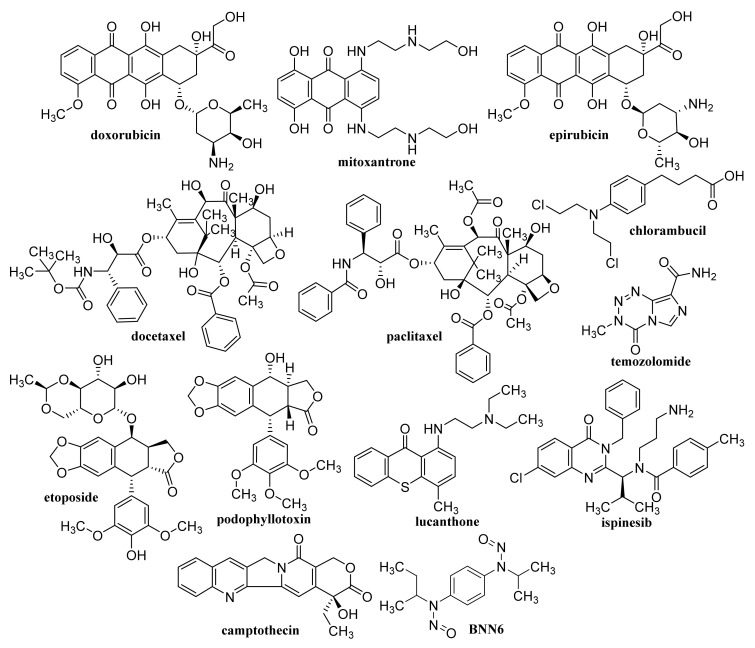
Selected drugs for cancer chemotherapy investigated for delivery using GR- and/or CNT-based nanoDDSs.

**Figure 4:**
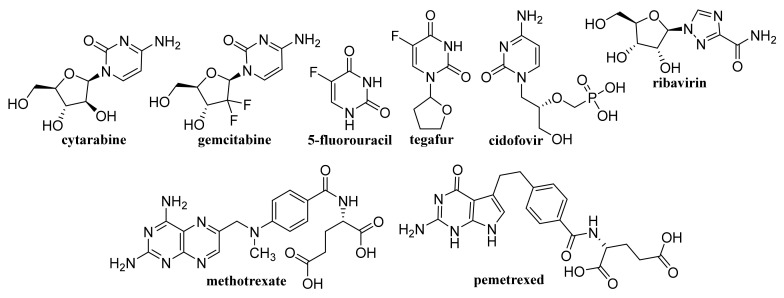
Selected cancerostatic and virostatic antimetabolites investigated for delivery using GR-based nanoDDSs.

GQD cross-linked carboxymethyl cellulose nanocomposite hydrogel showing a pH-sensitive swelling and degradation with improved tensile strength exhibited pH-sensitive doxorubicin (DOX, Figure 3) delivery behavior, enabling its use as a pH-triggered site-specific drug delivery system [130]. pH-responsive poly(d,l-lactide-co-glycolide) NPs coated with bovine serum albumin and encapsulating in their cores of GQDs and DOX, which showed superb blood compatibility, exhibited improved drug release in a mild acidic microenvironment, a dose- and time-dependent cytotoxicity to the HeLa cells, and contributed to a lower cancer cell viability [131]. GQDs loaded with cytarabine (Figure 4) and wrapped with chitosan (CS) gels to achieve the encapsulation of the loaded drug were characterized with improved fluorescent stability due to suppressed agglomeration of GQDs by the CS gels. The pH-sensitive release of cytarabine from this nanocarrier related to the hydrolysis of the amide linkage between GQDs and the drug in acidic medium [132]. GQD-modified poly(*N*,*N*-diethyl acrylamide) nanohydrogel with particle sizes 68.1–87.5 nm encapsulating DOX showed a considerably ameliorated drug release at temperatures of release media close to physiological temperatures. Based on the results of in vivo studies, in which this nanohydrogel was applied to mice with metastatic lung cancer, it can be considered as an intelligent drug carrier for melanoma cancer [133].

By co-encapsulation of GQDs and iron inside the core of an engineered ferritin nanocage derived from the archaeon *Archaeoglobus fulgidus*, GQD-iron complexes in the ferritin nanocages were formed; the nanocages exhibited high relaxivity in MRI, and strong fluorescence at low pH values and on MDA-MB-231. Insignificant cytotoxicity and high DOX loading capacity of 35% suggested that his nanocarrier has the potential to be used as a pH-responsive fluorophore, MRI agent, and drug nanocarrier in cancer diagnosis and therapy [134]. DOX-loaded microspheres fabricated by reaction of GQDs and magnetic carbon modified with 3-aminopropyltrimethoxysilane using a maltose disaccharide molecule covalently attached to a third generation triazine dendrimer (Fe_3_O_4_@C@TDGQDs) were non-toxic on the A549 cells and showed pH-dependent DOX release; such microspheres can be considered as a new safe and efficient vehicle for the delivery of cancer drugs [135]. GQDs-capped magnetic mesoporous SiO_2_ NPs with a particle size of 100 nm loaded with DOX released the drug at an acidic environment and efficiently generated heat to the hyperthermia temperature under an alternating magnetic field or by NIR radiation. At application of the combined chemo-magnetic hyperthermia therapy or chemo-photothermal therapy with the DOX-loaded magnetic mesoporous SiO_2_ NPs/GQDs nanoDDS, a remarkable synergistic effect in the killing of cancer cells was observed, exceeding the effects achieved using chemotherapy, magnetic hyperthermia or photothermal therapy alone [136]. Mesoporous SiO_2_ NPs capped with GQDs, which can serve as multifunctional drug carriers for photothermal and redox-responsive release, were designed by Gao et al. [137].

**Figure 5:**
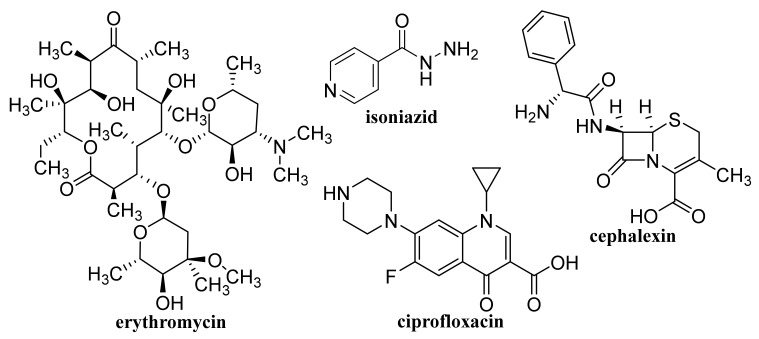
Antimicrobial drugs investigated for delivery using GR-based nanoDDSs.

A photo-responsive antibacterial composite system fabricated via loading both GQDs and erythromycin (Figure 5) into the hollow mesoporous SiO_2_ NPs exhibited an excellent therapeutic effect on the healing of wounds infected by bacteria *Escherichia coli* and *Staphylococcus aureus*, resulting in considerably reduced inflammatory factors in blood. The effective antimicrobial effect of the composite was due to ^1^O_2_ production by GQDs under light exposure, resulting in the breaking of the bacteria structure [138]. GQD-decorated hollow CuS NPs encapsulating DOX exhibited a pronounced near infrared (NIR)-triggered drug release within MDA-MB-231 cells after cellular uptake. The capping of GQDs on CuS NPs increased the conversion of light energy to heat to photothermally ablate the cancer cells under NIR laser irradiation, which can be utilized for combined photothermal-chemotherapy of cancer [139].

### 2.3. Functionalized GQDs

Hydroxylated GQDs (OH–GQDs) administered orally to C57BL/6J mice at a dose 5 mg/kg for 7 days caused significant intestinal injuries (e.g., enhanced intestinal permeability, shortened villi and crypt loss), strong drop in the number of Lgr5^+^ intestinal stem cells and inhibition of Ki67^+^ proliferative progenitor cells; OH–GQDs were found to upregulate both total and phosphorylated p53 and pronouncedly reduce the size of the surviving intestinal organoids in 3D organoid culture prepared using isolated crypts [140]. A smart FA-PEG-GQD-COOH drug vehicle showing encapsulation efficiency (EE) and drug-loading capacity of mitoxantrone (Figure 3) of 97.5 and 40.1%, respectively, was found to enter human cervical cancer cells predominantly via the macropinocytosis-dependent pathway, and the nanoformulation showed a strong antitumor efficiency and low systemic toxicity [141].

**Figure 6:**
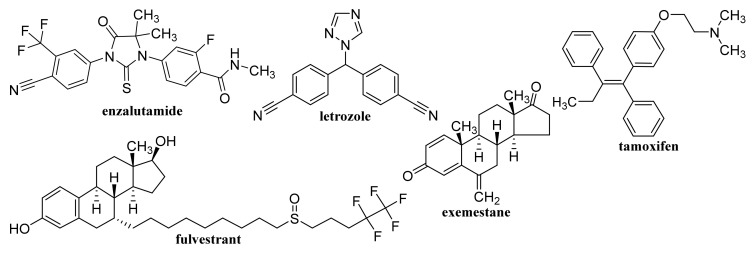
Drugs for treatment of gynecological and prostate cancer investigated for delivery using GR- and/or CNT-based nanoDDSs.

Non-functionalized GQDs at concentrations up to 250 μg/mL exhibited high biocompatibility on U87 human glioblastoma cells and primary cortical neurons, while dimethylformamide (DMF)-functionalized GQDs were biocompatible only at lower concentrations (50 and 100 μg/mL, respectively). At combined treatment with DOX both types of GQDs synergistically improved the efficacy of chemotherapy treatment, on U87 cells, particularly at application of 100 μg/mL DMF-GQDs. Improved DOX uptake by glioblastoma cells was associated with cell-specific changes in the membrane permeability of U87 cells due to GQDs and depended on GQD surface charge [142,143]. Xue et al. [144] reported that GQDs with an appropriate size may assist in the drug delivery process by reducing the translocation free energy permeating into the biomembrane. DOX-loaded water soluble GQDs were fabricated by acidic oxidation and exfoliation of MWCNTs, which were covalently linked to the tumor targeting module biotin (BTN). The GQDs and GQD-BTNs showed very low toxicity to A549 cells. Higher and delayed cell uptake was observed with DOX-loaded GQD-BTN compared to those estimated at treatment with free drug and with GQD-BTN. The delayed nuclear internalization of the drug associated with the removal of the drug from the nanoDDS was induced by the acidic environment of the cancer cells [145]. Enzalutamide (Figure 6)-loaded aminated GQDs cross-linked via disulfide bonds, which were additionally functionalized with a tumor-targeting peptide and polyethylene glycol (PEG), were characterized by high drug-loading efficiency via π-π electron interaction. The functionalized GQDs were rapidly internalized by castration-resistant prostate cancer cells via endocytosis, and showed good cancer-targeting ability and when loaded with enzalutamide, they inhibited the growth of C4-2B and LNCaP prostate cancer cell lines in vitro. Moreover, this GQDs nanocarrier showed a controlled drug release, an enhanced cancer-targeting ability and alleviated the side effects of drugs, suggesting that the formulation could be utilized in an intravenous therapy for this type of prostate cancer cell [146].

#### Nitrogen-Doped GQDs

Using a combined density functional theory and molecular dynamic approach, Vatanparast and Shariatinia [147] found that among nitrogen-doped GQDs (N-GQDs), the center N-GQDs exhibit improved performance in gemcitabine (GEM, Figure 4) drug delivery compared to that of pristine GQDs and edge N-GQDs. Drugs loaded on the surface of center N-GQDs was released advantageously in acidic environments of cancer tissues. In general, the drug release was facilitated at a perpendicular penetration of the nanocarrier into the membrane plane. N-GQDs with a particle size of approximately 10.9 ± 1.3 nm was found to cleave calf thymus DNA without any external agents, and showed superb antioxidant activity, and at 24 h incubation of cells with a dose of 200 μg/mL N-GQDs, the viability of A549 and MDA-MB-231 cells achieved 70% and the same cell viability was observed with NIH-3T3 cell lines using a dose of 150 μg/mL N-GQDs. Eudragit^®^ RS 100-coated N-GQDs loaded with EphA2-siRNA were rapidly internalized into A549 cells and could be applied for in situ tumor suppression via DNA and mRNA breakage [148]. In nanocomposites of N-GQDs incorporated onto the surface of the TiO_2_ NPs, the TiO_2_ NPs were situated on the 2D graphene nanosheet surface. The nanocomposites were not toxic to the MDA-MB-231 breast cancer cells at ≤0.1 mg/mL and at higher concentrations (0.5 and 1 mg/mL) showed pronouncedly lower cytotoxicity than the pristine TiO_2_ [149]. MgAl-layered double hydroxide-modified Mn_3_O_4_/N-GQD conjugated polyaniline nanocarrier fabricated for DOX delivery in breast cancer cells and exhibiting 90% DOX EE was characterized by slow drug release under normal physiological conditions, while pH-triggered drug release at low pH (corresponding to the extracellular tumor environment) achieved ca. 80% DOX. This DOX-loaded nanocarrier pronouncedly inhibited MCF-7 cells but did not affect the viability of human L929 cells and the nanocarrier showed excellent blood compatibility [150].

## 3. Graphene/Oxidized Graphene Nanoribbons and Nanoflakes

### 3.1. Graphene Nanoribbons

Graphene nanoribbons (GNRs) are narrow lengthened strips of single-layer GR characterized with an ultra-high surface area and unique cutting-edge electronic, thermal, mechanical, and optical properties associated with GR, whereby they can also effectively be uptaken by mammalian cells but are almost nontoxic to human health and the environment. The oxygenated derivatives of GNRs, graphene oxide (GO) nanoribbons, whether in form of their noncovalent or covalent modifications, can be used in various areas of biomedicinal applications, including drug delivery, anticancer, antimicrobial gene or photothermal therapy, imaging, bone regeneration, etc. [151,152,153]. Density functional study of zigzag GNR nanoribbon covalently functionalized with l-phenylalanine (fZGNR) and doped with boron near the edge carbon atom of fZGNR showed its increased chemical reactivity, reduced kinetic energy of electrons and higher stability in comparison with fZGNRs doped with boron away from the edge or in the center of the nanoribbon [154].

### 3.2. Oxidized Graphene Nanoribbons

Exposure of human neuroblastoma cell lines SK-N-BE(2) and SH-SY5Y to low concentrations of oxidized GNRs (O-GNRs) fabricated by oxidative unzipping of SWCNTs resulted in increased production of reactive oxygen species (ROS) and autophagy was induced in both neuroblastoma cell lines within a few hours of exposure, although growth arrest or cell death were not observed. Consequently, it can be assumed that GO nanoribbons could be used for therapeutic delivery to the brain tissue without an adverse impact on healthy cells [155]. O-GNRs can load large amounts of small-sized single-stranded or large-sized double-stranded genetic materials without additional functionalization with positively charged groups or other non-viral vectors. Complexes of O-GNR with plasmid DNA can be taken up into vesicular structures of dividing HeLa and HUVEC cells, released into the cell’s cytoplasm and enter the nucleus. A concentration- and time-dependent increase in gene delivery and gene transfection efficiencies up to 96–98% in these cells was observed with O-GNRs, which were loaded with enhanced green fluorescence protein plasmid or siRNA against glyceraldehyde-3-phosphate dehydrogenase [156]. Foreman et al. [157] designed an O-GNR-based platform for gene delivery of double-stranded DNA into mammalian cells. DNA loading and effective dispersion of O-GNRs was found to be stimulated by the presence of salts in medium; the surfactants did not affect the DNA-O-GNR complexes, which were not toxic to mammalian cells and the cargo DNA was expressed in the nucleus.

Low concentrations (<80 μg/mL) of O-GNRs noncovalently functionalized with 1,2-distearoyl-sn-glycero-3-phosphoethanolamine-*N*-[amino(polyethylene glycol)] (DSPE) were nontoxic to the components of the blood vascular system, and they only elicited deformation of red blood cells but hemolysis and histamine release from mast cells did not occur, although minor decrease in anti-inflammatory cytokine levels was observed. Consequently, such nanoformulations may be used for diagnostic and therapeutic applications in diseases of the circulatory system [158]. O-GNRs noncovalently functionalized with PEG-DSPE activated epidermal growth factor receptors (EGFRs) resulting in the generation of a predominantly dynamin-dependent macropinocytosis-like response leading to their pronounced uptake into cells with high EGFR expression. Improved uptake connected with the modulation of the activated EGFR by the viral protein E5 also showed cells with an integrated human papillomavirus genome [159]. Water-solubilized O-GNR-PEG-DSPEs showed a considerably different cytotoxicity profile than GR NPs prepared by oxidation of graphite and its mechanical exfoliation, they were uptaken by HeLa cells at a greater extent compared to other cell types (MCF-7 or SKBR3 cells) and already application of 10 μg/mL O-GNR-PEG-DSPEs to HeLa cells resulted in remarkable cell death up to 25% [160]. The uptake of PEG-DSPE-coated O-GNRs loaded with anti-tumor drug lucanthone (Figure 3), which is an endonuclease inhibitor of apurinic endonuclease-1 (APE-1), by glioblastoma multiforme (GBM) cell line U251 and APE-1-overexpressing U251 cell line exceeded 67% and 60%, respectively, post 24 h; cell death was necrotic, likely due to oxidative degradation of APE-1. Uptake of the formulation by MCF-7 and rat glial progenitor cells (CG-4) causing only minor or no toxic effects was considerably lower (38% and 29%, respectively) suggesting that nonspecific cytotoxicity to the surrounding healthy tissue was reduced [161]. DOX-loaded O-GNRs modified with phospholipid-PEG exhibited 6.7-fold lower IC_50_ values for chemo-photothermal therapy toward U87 glioma cells than values observed in traditional chemotherapy. O-GNRs modified with phospholipid-PEG did not show in vivo toxicity and were excreted from the body in urine, suggesting that this nanocarrier can improve the efficacy of the therapy and reduce the risk of side effects in the body [162].

Comparison of the cytotoxicity of O-GNRs (ca. 310 × 5000 nm) and GO nanoplatelets (ca. 100 × 100 nm) fabricated using the oxidative treatment of MWCNTs (ca. 100 × 5000 nm) and stacked GR nanofibers (ca. 100 × 5000 nm) confirmed considerably higher cytotoxicity of O-GNRs, which had a greater amount of COOH groups and a greater length. Moreover, cytotoxicity can be affected also by the type of carbon source used to prepare GO-based NPs [163].

### 3.3. Graphene/Graphene Oxide Nanoflakes

GR nanoflakes consist of a GR sheet ca. 30 nm in diameter with a pristine aromatic system and an edge terminated with COOH groups. Structural and dynamic properties in an aqueous solution are affected by their size and degree of oxidation. Although the curvature and roughness of GO flakes showing a relatively small size (3 × 3 nm) are not affected by their degree of oxidation, an increase in surface roughness with an increasing oxidation degree was observed at GO flakes, showing a greater size of 7 × 7 nm. On the other hand, the degree of oxidation did not influence the water dipole orientations past the first hydration layer, even though identifiable hydrophobic and hydrophilic patches on GO occurred due to the induction of a well-structured first hydration layer by oxygen functionalization [164]. GR nanoflakes were found to transport and stabilize the zinc phthalocyanine (ZnPc) molecule near the cell membrane for a longer time than the isolated ZnPc molecule, resulting in improved photodynamic therapy, whereby optical properties of ZnPc molecule interacting with GR nanoflakes were preserved both in a vacuum and water [165]. GR nanoflakes multi-functionalized with derivatives of (i) a peptide-based Glu-NH-C(O)-NH-Lys ligand, (ii) anti-mitotic drug (*R*)-ispinesib, (iii) the chelate desferrioxamine B, and (iv) an albumin-binding tag can extend pharmacokinetic half-life in vivo. (*R*)-Ispinesib (Figure 3)-loaded GR nanoflakes inhibited the kinesin spindle protein and induced G_2_/M-phase cell cycle arrest, while GR nanoflakes functionalized with the Glu-NH-C(O)-NH-Lys ligand showed specificity toward prostate-specific membrane antigen-expressing cells. Moreover, accumulation and retention of GR nanoflakes in background tissue was low, with rapid renal clearance, suggesting that they could be used in theranostic drug design [166]. ^99m^Tc-labeled ampicillin-loaded GO nanoflakes showed higher binding efficiencies to both *S. aureus* and *E. coli* than ^99m^Tc-labeled free drug [167]. Absorption of visible light in the range of 400–700 nm by GO nanoflakes and GO-nucleobase composites can be utilized in light-emitting devices [168].

**Figure 7:**
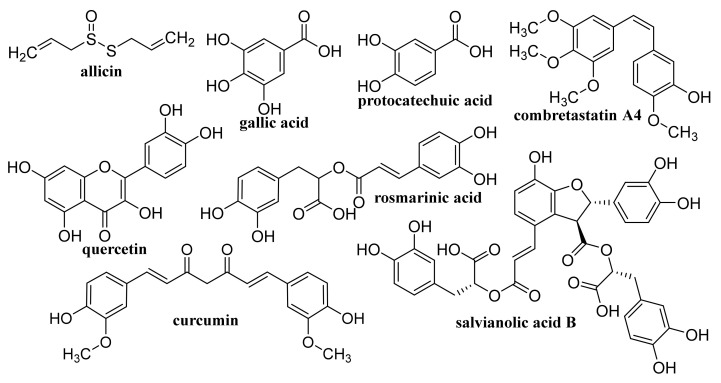
Discussed secondary metabolites of plants with antioxidant activity investigated for delivery using GR- and/or CNT-based nanoDDSs.

Rosmarinic acid (Figure 7)-loaded CS-GR NPs with a mean diameter 417.5 ± 18 nm showed strong antibacterial activity against *S. auresus* (IC_50_ 0.0038 ± 0.2 mg/mL) and after incorporation into Carbopol^®^ gel when they were tested in vivo for wound healing efficacy in Sprague Dawley rats, these NPs showed pronouncedly improved wound contraction, enhanced cell adhesion, epithelial migration, and high hydroxyproline content resulting in more rapid and more efficacious collagen synthesis than plain gel, pure drug and control [169].

## 4. Graphene Oxide

Biological processing and degradation of thin GO sheets by normal mammalian tissue was comprehensively discussed by Newman et al. [170] The splenic marginal zone is considered as the main site of GO bioaccumulation, whereby GO materials were not associated with detectable pathological consequences in the spleen. Applications of GO in regenerative dentistry, bone tissue engineering, drug delivery, improvement of physico-mechanical property of dental biomaterials, and oral cancer treatment were summarized by Nizami et al. [171] Composites of AgNPs, AuNPs, TiO_2_ NPs and Ag_2_O NPs with GO and reduced graphene oxide (rGO) showing large active surface areas enabling adhesion of organic and inorganic molecules suitable to be used in various biomedicinal applications including tissue regeneration, anticancer therapy, or bioimaging, were designed by Jagiello et al. [172] Recent progress of GO achieved by its surface modification resulting in significant improvement of its physicochemical properties and enabling its use as a potential vaccine carrier and adjuvant able to activate cellular and humoral immunity were summarized by Cao et al. [92] Functionalized GO as a promising material for delivery of chemotherapeutic drugs and cancer treatment was discussed by Sharma and Mondal [47]. GO used for biomedical applications can be also incorporated to hydrogels [93,94,95]. In general, recent developments in graphene and GO focused on their properties, fabrications and modifications were overviewed by Farjadian et al. [104] and varying degrees of oxidation can modify interactions of GO with proteins [173].

### 4.1. Unmodified GO

**Figure 8:**
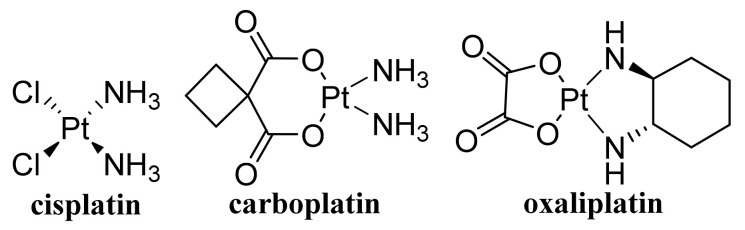
Structure Pt-based antineoplastics.

Improved efficacy of transdermal drug delivery with dissolvable polymeric microneedles achieved by the incorporation of a small amount of GO was demonstrated with transdermal delivery of the chemotherapeutic, HA15, to melanoma-bearing mouse models [174]. During loading of gallic acid (Figure 7), a natural chemotherapeutic agent showing antioxidant properties on GO, reduction of GO occurred resulting in a few-layer thin rGO and the formed biotinylated rGO nanocomposite was characterized with ameliorated targetability to A549 human lung carcinoma cells and enhanced cellular internalization efficiency, whereby it released the drug slowly at pH 7.4 in contrast to rapid drug release observed at a lower pH corresponding to the tumor microenvironment, thereby showing remarkable toxicity to A549 cells [175]. The physical adsorption of cisplatin (CDDP, Figure 8) on GO and rGO carriers was found to be an exergonic process in aqueous solution and due to the high hydrophilicity of the peripheral-COOH groups situated on the edge of the GO and rGO nanostructures by the adsorption of CDDP, its solubility and transport in water solutions can be improved [176]. Molecular dynamics simulation evaluating the mechanism of tegafur (Figure 4, prodrug of 5-fluorouracil) drug delivery by GO nanosheet across the cell membrane showed that GO was foremost spontaneously attracted to the cell membrane, whereby increasing formation of H-bonds between the O-containing groups of GO and lipid bilayer lightened a complete parallel orientation of the nanosheet; further, its partial insertion into the membrane and slow drug release from the GO nanosheet surface occurred near the cell membrane [177].

**Figure 9:**
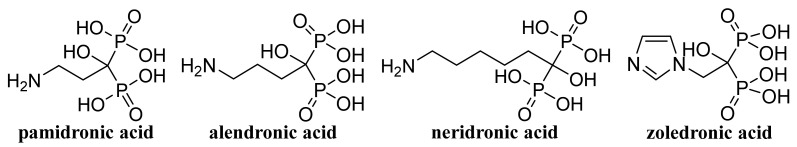
Mentioned drugs from group of bisphosphonates for treatment of bone diseases investigated for delivery using GR- and/or CNT-based nanoDDSs.

GO conjugated with zoledronic acid (ZOL, Figure 9), a third-generation bisphosphonate suitable for therapy of osteoporosis and metastasis, decreased the viability of MCF-7 breast cancer cells more than pure ZOL, but did not show a strong effect on the viability of bone marrow-derived mesenchymal stem cells (BM-MSCs). The conjugates were found to facilitate the mineralization of BM-MSC cells resulting in the formation of clusters around the cells; the most effective nanostructured ZOL-GO conjugates being fabricated using 50 μM ZOL and GO suspension of 2.91 ng/mL [178]. Modification of ciprofloxacin (Figure 5) with GO increased its cytotoxic potential in 786-0 (renal cancer) cells, and particularly T24 (human urinary bladder carcinoma) cells, as well as its apoptotic potential [179].

A needle-like GO nanocarrier obtained via conformational change of GO sheets using salt ions in the cell growth medium, was characterized with high surface area and satisfactory number of functional groups for DOX accumulation on the GO sheets. GO needles showed convenable biocompatibility at concentrations <100 μg/mL and after loading with DOX they showed ameliorated anticancer effectiveness compared to free DOX caused by improved cellular endocytosis of a 1D needle structure [180]. GO co-loaded with both DOX and the apoptotic agent antimiR-21 released rapidly both loaded compounds in cancer cells, resulting in effective destroying of cancer cells, whereby a low DOX dose was sufficient for the inhibition of MDA-MB-231 cells and antimiR-21 caused silencing of miR-21, upregulation of which is associated with numerous types of cancer [181].

### 4.2. Capped/Encapsulated GO

By incorporation of bio-based polymers into GO and rGO nanolayers, two-dimensional materials showing ionic conductivity, molecular transport, good mechanical properties, biocompatibility, and sustainability can be fabricated, which can be applied not only in biomedical engineering but also for efficient ionic and molecular separation technologies or for the construction of energy-related devices such as fuel cells and transistors [182]. Polylactic acid (PLA) scaffolds generated by 3D printing and reinforced by incorporation of GO showed improved thermomechanical and mechanical properties resulting in a 30% increase of the Young’s modulus at 0.3% GO content in the scaffold. Using MG-63 osteosarcoma cells it was found that PLA/GO scaffolds were biocompatible and stimulated cell proliferation and mineralization with higher effectiveness than pure PLA scaffolds [183]. Silicon contact lenses directly loaded with hyaluronic acid (HA) and GO exhibited a low burst with a sustained release of up to 96 h, and they were found to be safe in an ocular irritation study, showing potential for the improvement of tear fluid volume at managing ocular diseases like dry eye syndrome [184].

**Figure 10:**
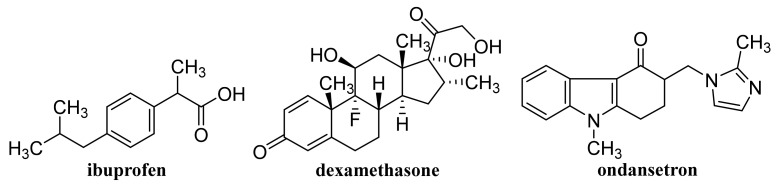
Mentioned anti-inflammatory drugs and antiemetics investigated for delivery using GR- and/or CNT-based nanoDDSs.

By freeze-drying of composites fabricated with integration of Na alginate (ALG) and GO using Ca^2+^ as the crosslinker, a nanohybrid carrier was formed showing electro- and pH-responsive release of entrapped methotrexate (MTX, Figure 4) due to the superb conductive properties of GO and Na ALG susceptibility to pH, whereby the release of MTX was controlled by Fickian diffusion [185]. Nanofibrous membrane fabricated using CS, polyvinyl alcohol and 0.1 wt% GO loaded with allicin (Figure 7) and showing pronounced hygroscopicity and moisture retention capacity exhibited good antibacterial activity against *S. aureus* also after 48 h, suggesting its suitability to be used as an antibacterial wound dressing [186]. Liang et al. [187] fabricated a GO/CS hybrid hydrogel with incorporated thin layer GO sheets modified with ZnO QDs, showing strong inhibition of *E. coli* and *S. aureus*; it exhibited combined effects of hyperthermia observed under the NIR irradiation of GO sheets, ROS generation, the release of Zn^2+^ ions from QDs at a low pH and the antibacterial activity, which can be used for wound healing. The relationship between GO size and its antibacterial activity against *Streptococcus mutans* was found to be parabolic; with the increasing GO size the cutting effect was reduced and the cell entrapment effect was enhanced, suggesting that GO size has an effect on its edge density and lateral dimension, and can affect its physical antibacterial mechanisms in different orientations and delineate its activity [188]. An electrospun nanocomposite of polycaprolactone (PCL)-based scaffolds containing GO nanosheets and dexamethasone (Figure 10), in which GO (thickness <1 nm) was uniformly distributed in PCL nanofibers, ameliorated the hydrophilicity, cell viability as well as pH changes compared to neat PCL scaffolds. Moreover, the nanocomposite exhibited a twofold increase in the osteogenic differentiation of mesenchymal stem cells and enhanced their biomineralization responses compared with the cells cultured in osteogenic differentiation medium, whereby the differentiation was stimulated more efficiently than the proliferation [189].

Max8 hydrogel was found to release GEM faster, achieving a 10-fold molar ratio to DOX. The composite prepared by suspension of selected NPs of DOX loaded on modified GO in a GEM/Max8 hydrogel matrix was effective against a triple negative breast cancer cell line, MDA-MB-231, showing more powerful synergism compared to the combination of both free drugs co-administered in form of solution [190].

### 4.3. Coated GO

A nanocomposite consisting of PEG-coated GO loaded with protocatechuic acid (Figure 7) and FA (Figure 1) exhibited in vitro uptake by HepG2 cells from 24 h and the migration ability of tumor cells was observed 48 h after treatment, suggesting that this nanocarrier can be used to improve the therapeutic efficacy of drugs [191]. Optimized PEGylated GO-PEG-cephalexin (Figure 5) nanoconjugate with drug EE of 69% and a loading capacity of 19% showed, after an initial burst release, a more sustained release over 96 h, achieving a cumulative release of 80% and exhibited both dose- and time-dependent antibacterial activity against Gram-positive as well as a Gram-negative bacteria. Its antibacterial activity against *S. aureus* and *B. cereus* expressed by minimum inhibitory concentration (MIC) values (7.8 and 3.9 μg/mL) was higher than that of the pure drug (MIC 10 μg/mL for both types of bacteria), suggesting that it can be used for treatment of infections caused by these bacteria [192].

**Figure 11:**
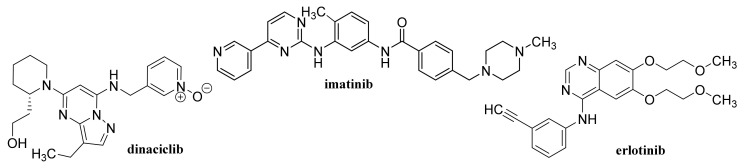
Mentioned inhibitors of kinases investigated for delivery using GR-based nanoDDSs.

GO was coated with 6-armed PEG loaded with a disulfide prodrug of podophyllotoxin (Figure 3) (DCM-S-PPT), which more effectively inhibited the proliferation of human cervical adenocarcinoma HeLa cells compared to human normal kidney 293T cells and showed better antitumor activity and the best tumor-targeting and specific drug release as well as lesser side effects than those of PPT, DCM-S-PPT and the complex of DCM-S-PPT with GO [193]. Docetaxel (Figure 3) conjugated via bonds to PEG chains of pegylated GO exhibited an excellent anticancer activity on DU-145 prostate cancer cell lines after 24, 48 and 72 h, indicating that PEGylated GO can serve as a suitable nanocarrier for the delivery of anticancer drugs to targeted tissues [194]. PEG-coated GO encapsulating erlotinib (Figure 11) strongly suppressed proliferation, migration, and invasion of nasopharyngeal carcinoma (NPC) cells and may be used as a potential therapeutic agent for treating NPC [195]. As a safe nanovehicle for DOX delivery in cancer therapy, a GO/PEG-b-poly(2-hydroxyethyl methacrylate-*g*-lactide)_2_ nanocomposite was also reported [196].

A system consisting of alendronic acid (Figure 9)-loaded collagen-GO sponges exhibited prolonged period of drug release in vitro, and they were found successfully to inhibit osteoclastogenesis of monocyte-macrophages and in vivo tests showed that sponges containing 0.05% (*w*/*v*) GO effectively enhanced the volume of newborn bone in the defect site in rats, suggesting the potential of such formulation for treatment of osteoporosis [197]. Thermosensitive injectable hydrogel containing a composite of (*N*-isopropylacrylamide)-based copolymer, GO and amounts of CS was found to enhance the deposition of minerals and the activity of alkaline phosphatase and to upregulate the expression of the Runt-related transcription factor 2 (key transcription factor associated with osteoblast differentiation) and osteocalcin in the human dental pulp stem cells (hDPSCs) cultivated in both the normal and osteogenic media, suggesting that it may be used as a constructing scaffold in bone tissue engineering for the transplantation of hDPSCs [198].

MCF-7 breast cancer cells treated with 8-hydroxyquinoline-coated GO showed pronouncedly increased cell deaths as well as considerably increased expression of P53, P21, and Bax genes and reduced expression of BCL2 genes compared to normal breast cells, MCF-10; as one of the mechanisms of this GO nanocomposite to induce cell death, induction of apoptosis in MCF-7 cells can be considered [199].

As a promising theranostic system for colon cancer MRI and targeting CT-26 colon cancer cells via folate receptors overexpressed on cancer cells a nanocomposite of GO integrated with polydopamine, bovine serum albumin, diethylenetriaminopentaacetic acid-manganese (II) contrast agent and 5-fluorouracil (5-FU, Figure 4), which was biocompatible and selectively distributed into the CT-26 tumors compared with liver and spleen, was designed by Foroushani et al. [200].

### 4.4. Functionalized GO

Biocompatible sulfonated GO nanohybrid scaffolds of CS with an interconnected uniform porous network structure showing a hydrophilic character and an improved mechanical strength to pure CS exhibited sustained release of tetracycline hydrochloride and were capable of supporting and proliferating MG63 osteoblast cells resulting in bone tissue regeneration. Using these nanohybrid scaffolds means that faster bone regeneration without any side effects can be achieved than with application of pure scaffolds [201]. The IC_50_ values related to cytotoxicity of etoposide (Figure 3) and etoposide-loaded carboxylated GO on hepatocellular carcinoma Hep-G2, were estimated as 6 ± 1.73 and 4 ± 0.11 μg/mL, respectively. The toxicity was caused via induction of the expression of the same seven apoptotic genes, although etoposide-loaded carboxylated GO induced apoptosis in Hep-G2 cells more efficiently than free etoposide [202]. Carboxylated GO conjugated with trimethyl CS hyaluronate NPs loaded with hypoxia-inducible factor (HIF)-1α-siRNA and dinaciclib (inhibitor of cyclin-dependent kinases (CDK1, CDK2, CDK5, and CDK9, Figure 11) considerably restrained the CDKs/HIF-1α and reduced the proliferation, migration, angiogenesis, and colony formation in tumor cells, suggesting that such dual drug/gene delivery in cancer treatment could be successfully used as an anticancer treatment strategy [203].

**Figure 12:**
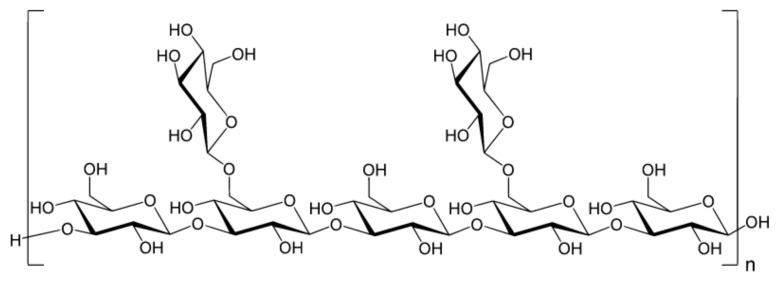
Structure of lentinan.

GO functionalized with lentinan (Figure 12), a polysaccharide isolated from the fruit body of shiitake mushroom (*Lentinula edodes*) known to have immunity-enhancing effects, potentiated antigen uptake in macrophages and ameliorated the efficiency of antigen application in vitro and showed a decreased release rate of ovalbumin, resulting in sustained long-term immune responses and augmented levels of IgG and IgG subtypes. Hence, GO-lentinan could be used as a superb carrier capable of eliciting a long-term immune memory response and enhance cellular and humoral immunity [204].

Temozolomide (Figure 3) loaded on FA-modified GO showed remarkable pH dependent drug release with sustained release properties in vitro and using a dose of 600 μg/mL for 72 h the inhibition of the growth of rat glioma cells achieved 91.72 ± 0.13% [205]. DOX-loaded GO and FA-functionalized GO showed a comparable but lower decrease of Ehrlich ascites carcinoma (EAC) cell viability in vitro than pure drug, and at treatment of EAC-bearing mice with GO/DOX, GO/FA/DOX and pure DOX the observed decreases of the total numbers of EAC by 79%, 97% and 97%, respectively, were observed, inducing cell cycle arrest at G_0_, G_1_, and S phase, respectively. Treatment with these conjugates resulted also in remarkable induction of apoptosis with different profiles on viable, early, and late apoptotic EAC cells. Hence, DOX-loaded FA-activated GO nanosheet can exhibit a comparable anticancer effect to free drug, even though the mechanisms of action differ each from other [206]. A novel gene vector, pegylated folate-modified GO/polyethylenimine (PEI) nanocomplexes with a mean size of 216.1 ± 2.457 nm, was found to rapidly escape from the lysosome and release the gene with entrapped siRNA gene, resulting in effective inhibition of the growth of ovarian cancer cells and such a nanoformulation can be used for treatment of folate receptor-positive ovarian tumors [207]. DOX-loaded GO, which was modified by Pluronic^®^ F68, FA and transferrin, allowing dual-targeting, showed controllable drug delivery performance with no toxicity, exhibited a higher inhibitory efficiency against human hepatocellular carcinoma SMMC-7721 cells than a single target drug delivery system without transferrin functionalization, and showed sustained release, being able to decrease the drug release rate in blood circulation over time and enhance drug concentration in or near a targeted tumor [208]. Nanohybrid prepared by functionalization of GO with Pluronic^®^ F127 molecules via non-covalent interaction practically did not show any toxicity against human astrocytes and human glioma (U251) cells. The DOX-loaded nanohybrid showing a drug loading efficiency of 83% with a loading capacity of 0.83 mg/mg induced a higher apoptosis of U251 cells compared to free DOX (12.27 ± 0.06 vs. 8.20 ± 0.06%), affected the mitogen-activated protein kinase signaling pathway and induced the intrinsic pathway of apoptosis for the activation of Caspase-3 in these cells [209].

Functionalization of GO with alanine resulted in a pronouncedly higher amount of loaded CDDP and drug-releasing rate compared to GO foams functionalized with cysteine and glycine due to larger surface area and pore volume of alanine-GO foam and a test with MCF-7 and HepG2 human cancer cell lines showed satisfactory biocompatibility and sustainable CDDP release up to 48 h [210]. GO@peptide hybrids fabricated through irreversible physical adsorption of the Ac-(GHHPH)_4_-NH_2_ peptide sequence, known to mimic the anti-angiogenic domain of the histidine-proline-rich glycoprotein, showed powerful toxicity to PC-3 prostate cells, effectively inhibited the cell migration, and inhibited the prostaglandin-mediated inflammatory process not only in PC-3 cells but also in human retinal endothelial cells. The mechanism of action of GO@peptide hybrids against the PC-3 cells consisted of GO-characteristic cell wrapping and mitochondrial perturbation [211]. Scaffolds fabricated using silk fibroin combined with functionalized GO loaded with a high amount of salvianolic acid B (Figure 7) continuously released the drug at maintaining its biological activity, pronouncedly stimulated the osteogenic differentiation of rat bone marrow mesenchymal stem cells, and strongly enhanced endothelial cell (EA-hy9.26) migration and tubulogenesis in vitro. Moreover, eight weeks after implantation of these scaffolds in a rat cranial defect model, the defect area showed more new bone and angiogenesis compared with the implantation of silk fibroin or the silk fibroin/GO scaffold [212].

Pourjavadi et al. [213] developed a carrier based on GO modified with poly(epichlorohydrin)-graft-hyperbranched polyglycerol containing pendant hydrazine groups capable of co-delivering DOX and curcumin (CUR, Figure 7). The release of both drugs was found to be pH-sensitive and a synergistic effect of co-delivery of DOX and CUR in the MCF-7 cancer was observed. Oxaliplatin (Figure 8)-loaded dual pH- and thermo-responsive nanocomposite, in which the GO surface was covalently functionalized with poly(*N*-vinylcaprolactam) and poly(glycolic acid), showed a higher cytotoxic effect to MCF-7 cells than free drugs or nanocomposites without a loaded drug [214].

### 4.5. Magnetic GO

Nanocomposites of CS-coated Fe_3_O_4_, SiO_2_ and GO (CS/Fe_3_O_4_/SiO_2_/GO) showing a loading capacity for CDDP of 84% and drug release of 76%, 88% and 71% at pH 7.4, 6.5, and 5.5, respectively, were designed by Abdel-Bary et al. [215] Magnetic GO fabricated via co-precipitation of Fe^3+^ and Fe^2+^ ions on GO surface and coated with mesoporous SiO_2_, which was loaded with IBU, released approximately 86% of the drug during the first 2 h. By encapsulation of this nanocomposite into carboxymethyl cellulose, sustained ibuprofen (IBU, Figure 10) release in 8 h can be achieved and such nanoformulation displayed the superparamagnetic properties [216]. Poly(glycidyl methacrylate)-coated magnetic GO nanocarrier was described as a highly efficient nanocarrier for magnetic- and pH-triggered delivery of DOX [217].

**Figure 13:**
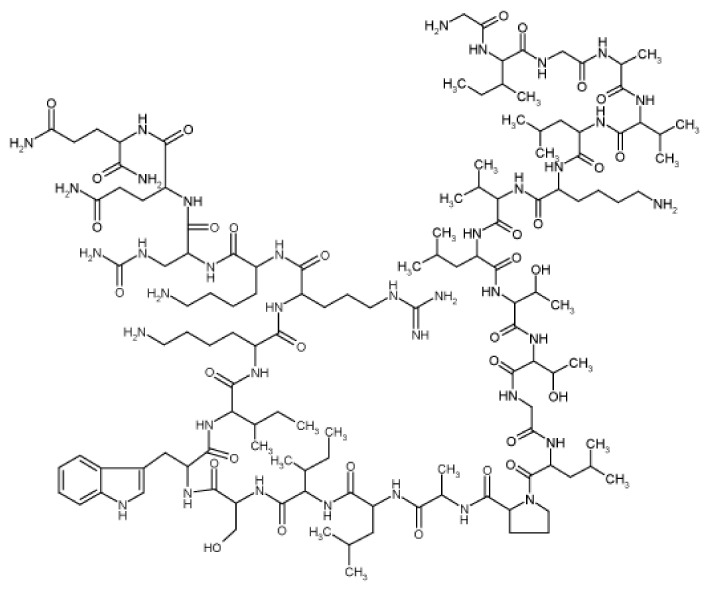
Structure of melittin—the main component of bee (*Apis mellifera*) venom.

Complexes of melittin (a main component of bee venom, Figure 13) with GO-based magnetic nanocomposites showed an enhanced inhibitory effect on HeLa cells and generated pore formation in the cell membrane resulting in cell lysis. Whereas PEGylated GO ensured protection of melittin, Fe_3_O_4_ NPs functioned as magnetic responders. Consequently, active drug was released up to 72 h and effectively inhibited the HeLa cells [218].

Nanosized conjugates of supraparamagnetic GO hybrid nanocomposite, GO/Fe_3_O_4_/FA, loaded with DOX exhibited excellent anti-tumor activity comparable with that of free DOX, impacting cell cycle and apoptosis of EAC breast cancer cells. Following stimulation of the conjugates with infrared radiation, the level of a cardiac biomarker, creatine kinase-MB, practically corresponded to normal levels, suggesting that by application of GO/Fe_3_O_4_/FA/DOX, formulation combined with brief hyperthermia anti-tumor effects with a lower cardiotoxic impact could be achieved [219]. The release of carboplatin (Figure 8) and oxaliplatin adsorbed on magnetic GO-Fe_3_O_4_-polyaniline NPs was pH-, dose-, and temperature-dependent. A more sensitive response to pH was observed with carboplatin, showing a higher release at pH 6.0 and 7.4 than at pH 8. At the higher temperature of 47 °C, slow release of the drug over a longer period was observed compared with small amounts of drug released at 27 °C [220]. A GO/Fe_3_O_4_ hybrid showing a temozolomide loading capacity of 6.47 ± 0.08 mg/mg and convenient drug release under slightly acidic conditions was not toxic to glioma C6 cells in the concentration range 40–120 μg/mL in vitro. On the other hand, temozolomide loaded on a GO/Fe_3_O_4_ hybrid showed an improved inhibitory effect on the proliferation of rat glioma C6 cells [221].

A multicomponent nanosupramolecular carrier composed of β-cyclodextrin (β-CD)/NiNP-modified GO (GONiCD) and mitochondrial ion-targeting peptide (MitP)-grafted HA showed a considerably improved DOX-loading capacity as well as drug-release efficiency under an alternating magnetic field (AMF) stimulus. At the application of AMF, DOX released from drug-loaded assemblies formed due to host–guest interaction between β-CD and the cyclohexyl groups on MitP, GONiCD and MitP-grafted HA during the DOX loading process can effectively target tumor mitochondria and damage both the mitochondria and the nuclei [222].

### 4.6. GO Used in Photoresponsive and Photothermal Therapy

Nacre-inspired multifunctional hierarchical and porous GO-CS-calcium silicate biomaterial was developed showing not only adequate strength, breathability, and water absorption but also superior photothermal antibacterial/antitumor and wound healing effects, which could be used in tissue engineering and regenerative medicine [223].

Enhanced delivery of DOX loaded onto the surface of GO to hepatic tumors in nude mice could be achieved using 20 kHz low-frequency ultrasound and microbubbles. Such treatment results in vascular endothelial cell wall rupture, widened endothelial cell gaps, black granules in the vascular lumen, interstitial erythrocyte leakage, and apparent apoptosis of tumor cells, whereby ultrasound cavitation damages tumor blood vessels and increases the release of GO-DOX stimulating apoptosis of tumor cells in nude mice [224]. At administration of DOX-loaded GO to cellular models of breast cancer, this nanoformulation was found to induce an immense intracellular drug release (followed by its nuclear accumulation) upon binding to the cell plasma membrane and exhibited considerably higher anticancer effectiveness than liposomal DOX [225].

The nanoplatform designed to overcome multidrug resistance consisting of R10 peptide conjugated to polyglycerol-covered nanoscale GO exhibited easier nuclear translocation due to R10 peptide; a laser-triggered release of the loaded DOX showed superb anticancer activity in vitro as well as in experiments performed in vivo. This nanoplatform was characterized with a high-loading capacity, controlled release of drugs, and photothermal properties [226]. NPs consisting of functional GO conjugated with PEG, FA and indocyanine green acting as photosensitizer fabricated for delivery of MutT homolog 1 (MTH1) inhibitor (6-(2,3-dichlorophenyl)-*N*^4^-methylpyrimidine- 2,4-diamine hydrochloride; TH287) and DOX applied in combined chemo-photodynamic therapy, inhibited the proliferation and migration of osteosarcoma cells, stimulated both apoptosis and autophagy by suppressing the MTH1 protein and enhanced ROS accumulation. Therefore, it can be assumed that ROS might contribute to endoplasmic reticulum stress and further induce apoptosis via the JNK/p53/p21 pathway. By suppressing MTH1 protein, “phenotypic lethality” could be induced and improved cellular sensitivity to ROS results in more efficient chemo-photodynamic therapy [227]. Following specific binding of aptamer to the MUC-1 receptor, its double strand separation occurs resulting in DOX release and fluorescence recovery (“on” state) at excitation with an excitation wavelength of 300 nm. Moreover, the platform responded selectively to tumor cells, showing higher toxicity against MUC-1-positive tumor cells (HT-29 and MCF-7) compared to MUC-1-negative cells (Hep-G2). A pH-responsive nanohybrid carrier was fabricated via chelating ZnO-dopamine on GO surface with a specific surface area of 37.16 m^2^/g and a DOX loading capacity up to 99.7% released the entrapped drug in the acidic environment for 14 days and exhibited a substantially higher toxicity against breast cancer cells T47D than against human mammary epithelial cells, MCF10A, as well as antimicrobial activity against both Gram-positive and Gram-negative bacteria [228].

GO-based nanosheets loaded with 5-FU and modified with GE11 (efficient ligand for EGFR) were able to transfer the drug into EGFR-overexpressing HCT-116 cells effectively, and after irradiation accelerated oxidation of glutathione in the tumor cells was observed, resulting in destruction of the intracellular redox balance after irradiation; in a subcutaneous colorectal cancer bearing mouse model, the nanocomposite exhibited 90% tumor inhibition [229].

NIR-responsive core-shell Au nanorods/mesoporous SiO_2_ NPs capped with PEGylated GO exhibited good photothermal stability in a physiological environment and acidic media as well as superb DOX loading efficiency and photothermal conversion efficiency (39.53%) showing potential to be used as a multifunctional platform for remotely controllable drug delivery [230]. Hybrid nanosheets fabricated by immobilization of Au nanorods onto the surface of PEGylated GO via polydopamine and showing an 86.16% DOX-loading capacity and a very low cytotoxicity to MCF-7 cells exhibited a pH/NIR-stimuli-responsive drug release, suggesting that they can be utilized as a superb platform for remotely triggered drug delivery [231]. A multifunctional cancer nanotheranostics system for non-invasive imaging, and targeted chemotherapy and tracing particular biomarkers based on PEGylated GO-AuNPs and specified with aptamer toward the mucin-1 (MUC-1)-positive tumor cells acting as an “on/off” fluorescence biosensor loaded with DOX was designed by Esmaeili et al. [232] A nanocomposite consisting of SiO_2_-based mesoporous Ti nanocarriers with photoactivable GO modified with a stealth polymer for delivery of imatinib, which can be used for NIR-triggered drug delivery and enhanced chemo-photothermal therapy was prepared by Gautam et al. [233] Drug release from this carrier achieved ca. 60% under light condition at pH 5 and the formulation efficiently converted NIR light into thermal energy (43.2 °C), which resulted in ROS generation. The combined effect of the chemotherapeutics imatinib (Figure 11) with the photothermal effect and ROS generated by GO was responsible for the considerable cytotoxic effect on colon cancer cells (HCT-116 and HT-29); pronounced accumulation of the composite in the tumor area and suppression of the tumor growth under NIR irradiation was observed in vitro and in vivo study.

Multifunctional methacrylate-modified gelatin/HA graft dopamine nanocomposite hydrogel containing an NIR-responsive β-CD-functionalized GO nanovehicle combined with BNN6 (*N*-(butan-2-yl)-*N*,*N*’-dinitroso-*N*’-(propan-2-yl)benzene-1,4-diamine, Figure 3), the NO donor, ameliorated collagen deposition and angiogenesis and stimulated wound healing in a mouse model of full-thickness skin repaired better than Aquacel Ag dressing, suggesting its suitability to be used for effective regeneration of bacteria-infected wounds [234].

### 4.7. Reduced GO

5-FU and CUR loaded CS/rGO nanocomposite showing drug EE >90% exhibited synergistic cytotoxicity and effectively inhibited the growth of HT-29 colon cancer cells with IC_50_ of 23.8 μg/mL [235]. 5-FU and CUR-loaded nanocomposites consisting of Pd nanospheres on CS/rGO exhibited sustained release of the drugs and inhibited the growth of human colon cancer cells (HT-29) [236]. PEGylated nanoceria decorated rGO nanocomposite exhibiting high DOX loading, pH-responsive drug release and a lesser harmful effect on normal cells than cancer cells as compared with free DOX and showed higher cytotoxicity on the cancer cells than the covalently conjugated rGO-PEG-DOX [237]. Optimized FA-functionalized gelatin-rGO formulation with particle sizes of 300 nm showing chlorambucil EE of 56% released 62.1% and 82% of the total bound drug at pH 5.4 and pH 1.2 respectively, while at pH 7.4 the released amount was 43.7% in the first 24 h. Using a drug-loaded gelatin-rGO nanocomposite, the % viability of human cervical adenocarcinoma cells at application of 500 μg/mL after 24 h achieved 28% compared to 11.7% observed with free drug after 24 h; pronouncedly lower cytotoxicity of chlorambucil-loaded nanocomposites expressed by the IC_50_ value compared to the free drug was estimated as well (86 vs. 125.9 μg/mL) [238]. Approximately 58.4%, 23.7%, and 16.2% of imatinib encapsulated in the nitrogen doped porous rGO-carboxymethyl cellulose nanocarrier was released at the pH 4, 7, and 9, respectively, after 20 h. Efficient drug loading at a drug to nanocarrier ratio of 1:1 (ca. 74% at pH 7.00; time: 3 h) was connected with the interaction of –OH and –COOH groups situated on the surface of the nanocarrier with imantib via H-binding and π-π stacking [239].

**Figure 14:**
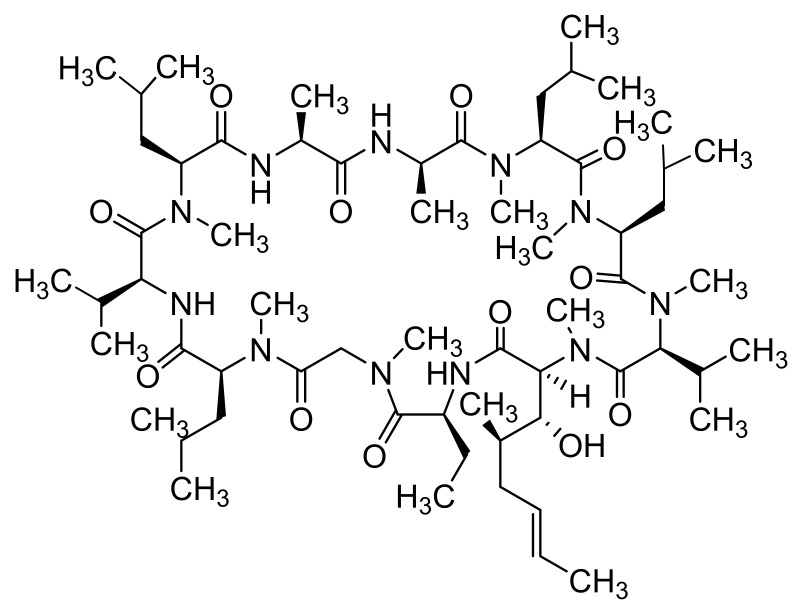
Structure of cyclosporine.

Quercetin (Figure 7)-loaded rGO-Fe_3_O_4_ nanocomposite stabilized using *Ganoderma lucidum* extract showing anticancer properties, which enhanced its water dispersibility and stability, was found to be cytotoxic to A549 cells. On the other hand, Pluronic^®^ F-127 introduced on the surface of the nanocomposite ensured a lower overall cytotoxicity of nanocomposite [240]. Promising transdermal delivery system based on ondansetron (Figure 10) loaded Pluronic^®^ F127 stabilized rGO hydrogel (2% Carbopol^®^ 940 base) able to enhance the bioavailability and ensure sustained release of the drug used to manage vertigo, nausea and vomiting was developed by Li et al. [241] Similarly, cyclosporine (a drug applied also in psoriasis treatment, Figure 14) incorporated in Pluronic^®^ F127 stabilized rGO hydrogel showed ameliorated permeation and retention, and the formulation was able to trap high amounts of the drug inside the skin tissue and reduced hyperplasia and tissue damage in psoriatic skin [242].

Cytocompatible, a fluorescent and superparamagnetic nanocomposite consisting of CS-coated rGO and implanted with Fe_3_O_4_ NPs achieving DOX loading of 0.448 mg/mL in vitro, was fabricated by Karthika et al. [243] Surface modification of Fe_3_O_4_ NPs with FA resulted in ameliorated uptake by A549 and MCF-7cancer cells and DOX release from this nanocomposite can be pH-triggered and controlled magnetically. Camptothecin (Figure 3)-loaded rGO decorated with magnetic NPs and cross-linked with a 4-hydroxycoumarin photosensitizer via an allylamine linker was found to be more toxic to MCF-7 cells than to normal fibroblast cell lines (WS-1). For powerful excitation of the photosensitizer needed for enhanced ROS production resulting in strong inhibition, apoptosis and death of MCF-7 cells, irradiation of this nanoformulation with a 365 nm laser (20 mW/cm^2^) for 3 min was sufficient. Moreover, loading of both camptothecin and photosensitizer on modified rGO also resulted in the synergistic anti-tumor efficiency in vivo exceeding that observed with monotherapy [244].

Irradiation of the CS-agarose thermogel incorporating rGO with near infrared (NIR) light caused a 3.8-fold higher temperature increase compared to hydrogel with incorporated GO and was able to reduce the viability of breast cancer cells to 60%. Further reduction of cancer cells’ viability to 34% was observed following incorporation of DOX and IBU drugs applied at an optimized ratio into the thermogel. Moreover, NIR laser irradiation of hydrogel resulted in its improved antibacterial activity as well [245].

## 5. Carbon Nanotubes

CNTs exhibit excellent properties such as strength, and high electrical and heat conductivity. Their uniqueness can be attributed to the bonding pattern presented between the atoms, which are very strong and also exhibit high extreme aspect ratios. Even so, CNTs, without any surface modifications, are generally cytotoxic to certain mammalian cells; following their functionalization, they become biocompatible and non-immunogenic [246]. Mohanta et al. [247] summarized current findings concerning in vitro and in vivo toxicity of CNTs in various organs of the body causing cellular toxicity. Due to the cage-like structure of CNTs, the delivered drugs can be isolated from the solvent medium. Both SWCNTs and MWCNTs have great potential to be used in breast cancer and increase diagnostic accuracy [248].

### 5.1. Single-Walled Carbon Nanotubes

The molecular interactions responsible for the adsorption of peptides and proteins on SWCNT surfaces, emphasizing the contributions from individual amino acids as well as secondary and tertiary protein structures and conformations, were analyzed by Antonucci et al. [249] Such a direct adsorption of proteins and peptides onto SWCNTs can be utilized for drug and gene delivery, in vivo imaging and targeting or cancer therapy.

#### 5.1.1. Unmodified SWCNTs

SWCNTs with diameters of 5–15 nm covalently filled with combretastatin A4 (CA4, Figure 7), an anticancer drug inducing cell apoptosis by inhibiting tubulin polymerization, showed ca. 90% release of the loaded drug over 50 h, exhibited pronounced increase in necrotic cells at the expense of the proportion of the apoptotic cells, and caused G_2_/M arrest, whereby at treatment with CA4-loaded SWCNTs, a greater proportion of cells was in the G_1_-phase in contrast to treatment with free CA4, when a higher proportion of cells in the S-phase was observed [250]. Translocation of bioactive molecules (e.g., asymmetric interfering RNA, single-stranded DNA, ubiquitin protein) through SWCNTs embedded in the 1-palmitoyl-2-oleoyl-5n-glycero-3-phosphocholine lipid membrane using fully atomistic molecular dynamic simulations was predicted by Sahoo et al. [251].

Zhang et al. [252] performed a molecular dynamics study on the configuration and arrangement of DOX in SWCNTs and found that they can be affected by drug concentration and by the diameter of the SWCNT. At relatively small SWCNT diameters, DOX molecules favored formation of a single-file helix inside the SWCNT, suggesting that by precise fabrication of SWCNTs showing specific diameters, controlled loading and release of a single drug molecule can be obtained, and an aggregated DOX structure in solution resulting in reduced chemotherapy dosage could be eliminated.

#### 5.1.2. Capped/Encapsulated/Coated SWCNTs

Using human lung adenocarcinoma (A549) cells, Singh et al. [253] estimated the anticancer potential of ALG- and CS-coated SWCNTs showing pH-dependent release of entrapped CUR. The nanocomposite of Ca-ALG and SWCNT modified by glucose using a ratio of 1:1 exhibited the fast release of entrapped CUR at pH 4.5 and pH 7.5 during the first few hours, followed by sustained release. The nanoformulation showed notable antibacterial activity against *Bacillus cereus* and *E. coli*, exceeding that of free CUR [254].

PEGylated and Tween-coated SWCNTs administered intravenously to BALB/c mice did not show a pronounced acute toxicity, even though in liver tissue increased expression of some proteins with antioxidant activity and detoxifying properties were observed as well [255]. PEG-modified composites of SWCNTs and polycationic and amphiphilic peptides H–(–Lys–Trp–Lys–Gly–)(7)–OH and H–(–Cys–Trp–Lys–Gly–)–OH showing uptake by A549 human lung adenocarcinoma epithelial cells were reported as drug and gene delivery carriers [256]. SWCNT-PEG-GEM conjugates showed a higher efficacy in suppressing tumor growth in B6 nude mice compared with SWCNT-GEM conjugate and free drugs and showed lower cytotoxicity to A549 cells and MIA PaCa-2 (human pancreatic carcinoma) cells than SWCNT-GEM conjugate [257]. RGD (arginine-glycine-aspartic acid)-decorated CS-coated SWCNTs loaded with docetaxel showed a considerably higher drug release at pH 5.0 (68%) than at pH 7.4 (49%) and exhibited pronounced inhibition of the growth of A549 tumor cells in vitro, and high cellular uptake in A549 cells in vitro, mainly through clathrin and caveolae-mediated endocytosis, whereby they also caused an effective inhibition of tumor growth of A549 cell-bearing nude mice in vivo [258].

Karnati and Wang [259] performed molecular dynamic simulations focused on co-loading and release of DOX and paclitaxel (PTX, Figure 3) using CS-coated SWCNTs and found that the drugs prefer binding to the sidewall of the SWCNT rather than binding with CS, even though reduced binding of DOX and PTX with the sidewall was estimated compared to uncoated SWCNTs. The protonation of CS and drug molecules relates to impairment of interaction between DOX/PTX and coated SWCNTs, resulting in displacement of the drug molecules, inducing the release of the drugs.

#### 5.1.3. Functionalized SWCNTs

Using density functional theory calculations, Pinto and Magalhaes [260] characterized covalent tip-functionalization of SWCNTs with –CH_2_–COOH groups that create pH-sensitive molecular gates and found that their responses to pH changes relate to the extent of protonation-dependent alternations in the noncovalent interactions between functionalized groups resulting in conformational changes. Thus, functionalized SWCNTs can be applied as efficient drug delivery systems. Comparison of the behavior of pristine armchair-type SWCNTs created with the help of software named AVOGADRO and those functionalized with –NH_2_, –COOH and –OH groups was performed by Garg and Negi [261] using the software MOPAC. They found variation in the charge value of SWCNT when these groups were brought in close proximity of the CNT and stated that the end-functionalized CNT can be used as an enzymatic nanomotor able to deliver an enzyme molecule inside the human body. Gajewska et al. [262] performed noncovalent as well as covalent functionalization of SWCNTs with the phosphono-perfluorophenylglycine analogue, a fluorinated phosphonate analogue of phenylglycine. The presence of perfluorinated phenyl rings enables the introduction of miscellaneous nucleophiles via nucleophilic aromatic substitution reactions to the functionalized SWCNTs, resulting in new materials, which could be used for drug delivery. Comparison of the affinity of three anticancer drugs, exemestane (Figure 6), letrozole (Figure 6) and fulvestrant (Figure 6), to SWCNTs using a molecular dynamic simulation study showed that the strongest affinity exhibited highly hydrophobic fulvestrant. Fulvestrant was strongly bound also to glycine-functionalized SWCNTs due to more active sites enabling H-bond formation between the drug and the functional groups of SWCNTs [263].

Complexes formed by non-covalently coated HA on NH_2_-SWCNTs via simple electrostatic adsorption and exhibiting DOX loading of 81.5%, showed faster pH-triggered drug release at pH 5.5 than at pH 7.4, which was favorable for intracellular drug release. Such a nanoformulation exhibited considerably improved intracellular delivery of DOX in CD44-overexpressing MDA-MB-231 cells, inhibited proliferation, and induced apoptosis of cells to a greater extent than DOX-loaded unmodified SWCNTs and was able remarkably to inhibit the migration of MDA-MB-231 cells [264]. SWCNTs functionalized on the surface with polyampholytic-alternating polymers having furfuryl amine and 3-(dimethylamino)-1-propylamine as functional groups showed a DOX-loading content up to 150 wt.% and exhibited a burst drug release at pH 5.5, corresponding to that in the microenvironment of tumor cells, while the DOX release rate at pH 7.4 was lower. Whereas such SWCNT hybrids were highly cytotoxic to HeLa cancer cells, their cytotoxicity against human embryonic kidney 293 (HEK293) cells was low [265].

The system consisting of SWCNTs pre-functionalized covalently with PTX and FA was found to be suitable for targeted delivery of PTX [266]. The hybrid silk hydrogel composite consisting of silk protein and DOX-loaded FA-functionalized SWCNTs showing stimulated on-demand DOX release after intermittent exposure of NIR light and active targeting to FA receptor-positive cancer cells, which can be injected to the targeted site or applied as intratumoral implantation, can represent a depot for anticancer drug-loaded NPs and reduce systemic side effects [267].

Formononetin-loaded hydroxypropyl-β-CD-modified carboxylated SWCNTs showing a drug entrapment efficiency and a loading capacity of 88.66% and 8.43%, respectively, exhibited slow and sustained formononetin release and higher antitumor activity in vitro compared to the free drug [268]. Carboxylate and bisphosphonate functionalized SWCNTs radiolabeled with ^99m^Tc were uptaken by tumors of Ehrlich tumor-bearing Swiss mice, uptake by bisphosphonated SWCNT being higher. The acute toxicity study performed in healthy Swiss mice confirmed that both types of SWCNTs did not exhibit hematological, hepatic, or renal toxicity [269].

#### 5.1.4. Magnetic SWCNTs

Adsorption of 5-FU onto either pristine or chloromethylated semiconductive (8.0) SWCNTs was found to be physical and exothermal and so it does not affect the structure of the drug. Chloromethylated semiconductive (8.0) SWCNTs were found to be very suitable as 5-FU carriers, which are able safely to deliver the drug to the target by application of magnetic fields. A combination of these functionalized (8.0) SWCNTs and 5-FU is a ferromagnetic bipolar semiconductor, which can be utilized to guide the drug also via an external electric field [270]. Al Faraj et al. [271] developed improved targeting of DOX-loaded SWCNTs to metastatic regions, the therapeutic effect of which was enhanced when high-energy flexible magnets were specifically positioned over the metastatic tumor sites in the lungs. Using an MRI subtle monitoring of a nanocarrier, biodistribution in the abdominal organs, preferential homing towards the metastatic sites, as well as an improved therapeutic effect was ensured. Multifunctional magnetofluorescent PEG 2000 *N*-modified Fe_3_O_4_@carbon QD-coated SWCNTs loaded with DOX and conjugated with a sgc8c aptamer induced photodynamic and photothermal ablation of the targeted lung cancer cells and after irradiation with pH/NIR, laser rapid DOX release was observed, suggesting that the formulation can be used for combining cancer photothermal therapy, photodynamic therapy, and chemotherapy [272].

### 5.2. Multi-Walled Carbon Nanotubes

Sheikhpour et al. [273] critically overviewed applications of CNTs in the diagnosis and treatment of lung cancer, emphasizing that their side effects manifested during therapy such as inflammation, fibrosis, and carcinogenesis can be partially reduced by functionalization of CNTs to proper dimensions, such as a longer length, a greater width, and a greater curvature. At studying the effect of MWCNTs on the toxicity of cytotoxic compounds in macrophage (RAW 264.7), lung epithelial (A549), and breast cancer (MCF-7) cell lines, it was found that hydrophilicity/lipophilicity of the compounds was determinant. The dependence of log *P* vs. cytotoxic activity showed a quasi-parabolic course, the highest value being observed at log *P* close to 1 and the degree of cellular uptake of MWCNTs affected the cytotoxicity of drug/MWCNT combinations, suggesting that MWCNTs function as a “Trojan horse”, ensuring improved intracellular delivery of drugs leading to increased cytotoxic activity [274]. Nahle et al. [275] compared in vitro responses of rat alveolar NR8383 cells to pristine, anionic, and cationic MWCNTs using cytotoxicity assays, transcriptomics, and proteomics. Whereas ribosomal protein translation, cytoskeleton arrangement and induction of the pro-inflammatory response was observed with all MWCNTs, induction of mTOR (mammalian target of rapamycin) signaling pathway in conjunction with increased Lamtor gene expression was determined only with functionalized MWCNTs. However, cationic MWCNTs also activated the transcription factor EB and induced autophagy, while application of anionic MWCNT altered eukaryotic translation initiation factor 4 and phosphoprotein 70 ribosomal protein S6 kinase signaling pathway and caused upregulation of TLR2 gene expression. Hence, even though MWCNTs toxicity is reflected primarily in inflammation, their toxicity mechanisms are functionalization dependent.

#### 5.2.1. Unmodified MWCNTs

Dipyridamole-loaded MWCNTs showed an increased drug dissolution rate, which decreased with increases in the drug-loading rate of carriers. Moreover, with increasing drug loading, the dipyridamole in the MWCNTs changed its form from an amorphous to a crystalline state [105]. Bisphosphonate (alendronic, neridronic and pamidronic acids, see Figure 9)-conjugated MWCNTs showed pH-dependent drug release, which was faster at lower pH values. Neridronate-conjugated MWCNTs inhibited MCF-7 cancer cell death more compared to free alendronic acid, which can relate to the selective targeting of cancer cells by MWCNTs. On the other hand, pamidronate–MWCNTs conjugate suppressed the strong anticancer activity of free drugs [276]. Isoniazid (Figure 6)-conjugated MWCNTs showed pronouncedly improved antibacterial activity against standard strain of *Mycobacterium tuberculosis* H37Rv as well as against a strain sensitive to isoniazid compared to that of pure drug, a lethal effect being achieved at considerably lower doses (1/16 for sensitive strain and 1/32 for H_37_R) than at treatment with pure isoniazid. This can relate to better penetration of nanoformulation to the bacterial membrane, and higher delivery of isoniazid at lower doses, resulting in reduced bacterial resistance toward the usual form of antibiotics [277].

Treatment of pancreatic cells PANC-1 with MWCNTs co-loaded with pemetrexed (Figure 4) and quercetin using a dose of 25 μg/mL MWCNTs, 20 μg/mL pemetrexed and 2 μg/mL quercetin caused not only pronounced alterations of cell morphology but also reduced the cell viability (<60%) and resulted in higher intracellular ROS levels compared to treatments with single-nanoDDSs. On the other hand, the cytotoxic effects of single- and double-drug nanoDDSs in MDA-MB-231 breast cancer cells were comparable and less pronounced [278]. MWCNTs containing iron used as a catalyst during production and MWCNTs from which all iron was removed in a post-production heat treatment (leading to a considerably decreased number of surface defects) caused dose- and time-dependent decrease in the viability of A549 lung epithelial cells and HepG2 hepatocytes and increased lactate dehydrogenase leakage, suggesting a reduced membrane integrity. The comparison of both MWCNT types showed that heat-treated MWCNTs caused pronouncedly enhanced ROS production, resulting in a more negative impact on cells and induced a dose-dependent cell cycle arrest in A549 cells [279].

#### 5.2.2. Capped/Encapsulated/Coated MWCNTs

ALG-CS/MWCNTs (surface area: 7.854 m^2^/g; Youngs modulus: 249.17 N/mm) showing thermal stability up to 570 °C encapsulating IBU with 88% EE exhibited a prolonged and sustained drug release (68% in 6 h). The release of drug from the nanocomposite was greater in simulated body fluid than in simulated gastric fluid; greater temperature stimulated desorption of the drug from the nanocarrier, while increasing crosslinking and charge density of the nanocomposite reduced it [280]. MWCNTs/gelatin-CS nanocomposite films incorporating ciprofloxacin (Figure 5) showed sustained drug release after a sharp release in the first hour and exhibited higher antibacterial activities than gelatin-CS composite films loaded with antibiotic, which did not contain MWCNTs [281].

Dexamethasone-loaded, PEGylated, vertically aligned MWCNTs showing improved fast and prolonged release and releasing 55%, 65% and 95% of drug at pH of 7.4, 6.5 and 5.5, respectively, and lower toxicity in PC-12 cells in a wide concentration range (20–20,000 μg/mL) could be applied in treatment of ischemic stroke [282]. Enteric-coated carboplatin-loaded PEGylated MWCNTs showing EE of 71.58% did not exhibit a pronouncedly adverse impact on the viability of MDA-MB-231 cells and showed pH-responsive drug activity in a sustained manner, especially at simulated intestinal fluid conditions (pH 6.8). PEGylated MWCNTs showed potential to be used as nanocarriers for metronomic chemotherapeutic drugs, showing low oral bioavailability and pronounced anticancer effects because they enable chronic, equally spaced administration of low drug doses without extended rest periods. Even though using such low-dose chemotherapy did not result in complete tumor eradication, it can diminish the tumor burden as much as possible and maintain it over time [283].

Cidofovir (Figure 4)-loaded MWCNTs modified with cyclodextrins and branched PEI and doped with Rhodamine crossed the cell membrane via the clathrin-dependent pathway and co-localized in the lysosomal compartment; the powerful escape of drug from lysosome and its release close to the nuclear region induced the antiviral activity [284]. MWCNTs conjugated with PEI, an antiviral drug ribavirin (Figure 4) and the pearl gentian grouper nervous necrosis virus (PGNNV)-specific nanobody showed an increasing distribution in PGNNV-infected cells, powerful anti-PGNNV ability both in vitro and in vivo and such formulations can be an effective tool for virus-induced central nervous system disease targeted therapy [285]. PCL/poly(*N*-vinyl-2-pyrrolidone) (PVP) core-shell nanofibers showing mean diameter 300–400 nm and containing MWCNTs and 5-FU loaded in the core of these nanofibers showed sustained and prolonged drug release (up to 85% after 528 h) and cytotoxicity against HeLa cells (50.35% after 72 h), suggesting that this drug delivery system can be applied in post-surgical delivery of anticancer drugs [286].

Tri-stimuli-responsive MWCNTs covered by mesoporous SiO_2_ graft poly(*N*-isopropylacrylamide-block-poly(2-(4-formylbenzoyloxy) ethyl methacrylate) via disulfide linkages loaded with DOX showed the optimal release behavior in cancer environments compared with that in normal cells upon simultaneous triggering by pH-, temperature-, and reductant stimuli, and was able to function as an efficient gatekeeper to control the mesopore on-off and thus to modulate drug release. Moreover, the nanocomposite showed low toxicity, and the toxicity of the drug loaded on these materials corresponded to that of free DOX [287]. DOX formulated with TiO_2_-AuNPs-decorated MWCNTs showed better efficiency against A549 and MCF-7 cancer cell lines compared with the free drug, whereby the drug releasing capacity of 90.66% for 10 h was observed [288].

#### 5.2.3. Functionalized MWCNTs

Carboxy- and amino-functionalized MWCNTs, which were water soluble and biocompatible, caused pronouncedly improved cellular toxicity and prooxidant potential in HEK 293 cells compared to pristine MWCNTs, whereby carboxy-functionalized MWCNTs exhibited better tolerance to HEK 293 cells than the amino-functionalized ones. Reduced toxicity of functionalized MWCNTs can be attributed to changes in their agglomeration and to the surface charge [289]. Carboxylated multi-walled carbon nanotubes (MWCNT-COOH) functionalized with MTX, FA and PEI (MWCNT–MTX–PEI–FA) showed a reduced drug release rate compared to MWCNT-MTX (IC_50_: 9.89 ± 0.38 vs. 16.98 ± 1.07 μg/mL) due to the presence of PEI acting as a barrier. Even though, in the presence of an infrared laser the toxicities of high concentrations of MWCNT–MTX–PEI–FA to MCF-7 cells were comparable with those of MWCNT-MTX, they triggered the death of cancer cells by 55.11 ± 1.97%, compared to 49.64 ± 2.44% observed with MWCNT–MTX [290]. A smart nanoDDS consisting of quercetin, a mild P-gp efflux inhibitor, adsorbed on the nanoconjugate of *N*-desmethyl tamoxifen (Figure 6) with MWCNT–COOH mediated by tetraethylene glycol linker was prepared by Kumar et al. [291] At physiological conditions, the in vitro release of *N*-desmethyl tamoxifen from the conjugate was negligible compared with the free drug; however, it was considerably enhanced at an acidic pH corresponding to the microenvironment of cancer cells. This hemocompatible nanoformulation showed improved cellular uptake in drug-resistant MDA-MB-231 cells and in the systemic circulation of rodents the bioavailavility of the drug exceeded that observed with the free drug. Cellular viability of MDA-MB-231 cells treated with 2.52 μg/mL CDDP and 4 μg/mL of CDDP-loaded MWCNT–COOH was reduced by ca. 40% after 48 h of exposure despite the high oxidative stress generated by MWCNTs, which started with 24 h but after 48 h ROS levels decreased due to activation of the antioxidant defense. In cells exposed to MWCNT–COOH–CDDP, considerably reduced expression of caspase-3 and p53 and down-regulation of NF-κB was observed after 48 h; this system stimulated apoptosis escape and was not capable of overcoming the triple negative breast cancer cell resistance [292]. FA-ethylene diamine (ED)-anchored MWCNT–COOH covalently grafted with DOX via π-π stacking interaction showed strong in vitro anticancer activity on MCF-7 cells and excellent targeting specificity via overexpressed folate receptor but lower cytotoxicty [293]. pH-Responsive release at low pH values, targeting and causing strong growth inhibition of tumor cells overexpressing FA receptors, exhibited also nanocomplexes of DOX and multifunctional FA-bound MWCNTs fabricated using MWCNT–COOH covalently conjugated with PEI and subsequently stepwise modified with FA, fluorescein isothiocyanate and acetic anhydride/triethylamine. In vivo experiments showed that beside enhanced inhibition of tumor growth, these nanocomplexes were able to reduce the side effects of the free drug as well [294].

GEM-loaded HA conjugated-PEGylated MWCNTs exhibited a faster drug release rate at pH 5.3 compared to pH 7.4, which was followed by a sustained release pattern, and compared to free drug, they showed less hemolytic toxicity (7.73 ± 0.4% vs. 18.71 ± 0.44%) and higher cytotoxicity against HT-29 colon cancer cells. In vivo application of this nanoformulation to tumor-bearing Sprague Dawley rats caused a more pronounced reduction of tumor volume than free drug and increased survival rate without appreciable loss in body weight [295]. DOX-loaded MWCNTs functionalized with HA and α-tocopheryl succinate showed enhanced cellular uptake and anticancer-therapeutic activity against CD44 receptors overexpressing triple-negative breast cancer cells (MDA-MB-231) suggesting that they are suitable to be used in safe, and effective tumor-targeted chemotherapy [296].

For efficacious delivery of tumor antigens to dendritic cells (DCs) and triggering a powerful anti-tumor immune response, MWCNTs functionalized with mannose, which can specifically bind to the mannose receptor on the DC membrane, were fabricated using ovalbumin as a model antigen, capable of adsorption on these modified MWCNTs. The formed biocompatible complex was suitable for satisfactory antigen delivery and induction of DCs maturation and cytokine release in vitro and could be used for therapeutic purposes [297]. The surface functionalization with the bovine-milk-derived protein, succinylated β-lactoglobuline, can be considered as a cost-effective alternative to the PEG-based CNTs, leading to considerable amelioration of the biocompatibility and dispersion stability of CNTs, whereby MWCNTs functionalized with this protein were able to improve the IC_50_ values by ca. 5–6-fold compared to pristine MWCNTs in various cell lines [298].

#### 5.2.4. Magnetic MWCNTs

Epirubicin (Figure 3)-loaded magnetic MWCNTs containing Fe_3_O_4_ NPs showing sustained release and prolonged drug retention and better antitumor activity than free epirubicin in vitro and in vivo than the free drug can be considered as effective intravesical instillation agents for bladder cancer therapy [299]. DOX-loaded MWCNTs decorated with Fe_3_O_4_ NPs exhibited sustained drug release and an extended drug release time, enabling reduced dosing frequency, they inhibited growth of bacteria (*E. coli* and *Bacillus subtilis*) more efficiently, even at a lower concentration of drug loading, and can be directed by magnetism, showed ameliorated bioavailability and were able to exhibit targeted and prompt cellular uptake, resulting in remarkably improved drug efficiency [300].

#### 5.2.5. MWCNTs Used in Photoresponsive and Photothermal Therapy

MWCNTs with a dense coating of phospholipid PEG exhibiting minute nonspecific cell interactions but immense intercellular diffusion, chemically modified with an anti-P-glycoprotein (Pgp) antibody showed highly Pgp-specific cellular uptake, were strongly cytotoxic to multidrug-resistant (MDR) cancer cells upon photoirradiation but they were not toxic and phototoxic to cells, which do not express Pgp, suggesting their suitability for successful therapy of MDR cancers [301]. Comparison of cytotoxicity of MWCNT–COOH functionalized with MTX (MWCNT–MTX), and ED (MWCNT–MTX–ED) showed higher cytotoxicity of MWCNT–ED–MTX and the anticancer effects of both conjugates at application of infrared laser radiation on the MCF-7 cells were comparable or higher than without laser application, suggesting a strong photothermal effect based on the conversion of absorbed laser radiation by MWCNT to heat, inducing cancer cell death [302]. The cell inhibition rate and apoptosis rate of MCF-7 cells treated with tamoxifen-loaded lentinan-functionalized MWCNTs at application of NIR irradiation were by 67.1% and 66.5% higher than those observed with equivalent free drug dose with NIR irradiation, which can be attributed to the synergistic function of chemotherapy and photothermal ablation under application of NIR laser [303].

## 6. Conclusions

In today’s over-technological world full of various stressors, living in a polluted environment, food substitutes and a sedentary way of working, the proportion of civilization diseases is rising sharply, and a worrying trend of exponential growth in cancer can be seen. Despite appeals from various organizations, including the WHO, attempts to reverse this trend have been rather unsuccessful, and so scientists have to develop new drugs for the increasingly common cardiovascular, metabolic, neurodegenerative, locomotor, and cancer diseases. In addition to new drugs, reformulating existing drugs into new drug formulations providing modified release and/or targeted delivery is increasingly used. nanoDDSs are suitable for this purpose, especially for the delivery of drugs with a narrow therapeutic window (high systemic toxicity). In addition to vesicular and polymeric nanosystems, carbon-based nanosystems, mainly those based on graphene and carbon nanotubes, are also used. They are easily functionalized and used as nanoDDSs with insignificant cytotoxicity. Moreover, these materials can be advantageously used simultaneously as diagnostics, for direct therapy (thermal therapy, thermal tumor ablation, photodynamic therapy), or as tissue repair materials. Although these systems are predominantly in preclinical testing or experimental use, these carbon-based nanosystems represent a promising and growing group of nanomaterials with great potential for application in biomedical fields.

## Data Availability

Data sharing is not applicable to this article.

## Abbreviations

ALG (alginate);

AMF (alternating magnetic field);

APE-1 (apurinic endonuclease-1);

API (active pharmaceutical ingredient);

BBB (blood-brain barrier);

BM-MSCs (bone marrow-derived mesenchymal stem cells;

BTN (biotin);

β-CD (β-cyclodextrin);

CDDP (cisplatin);

CDK (cyclin-dependent kinase);

CNTs (carbon nanotubes);

CS (chitosan);

CUR (curcumin);

DCs (dendritic cells);

DCM-S-PPT (disulfide prodrug of podophyllotoxin);

DMF (dimethylformamide);

DOX (doxorubicin);

DSPE (1,2-distearoyl-sn-glycero-3-phosphoethanolamine-*N*-[amino(polyethylene glycol)]);

EAC (Ehrlich ascites carcinoma);

ED (ethylene diamine);

EE (encapsulation efficiency);

EGFR (epidermal growth factor receptors);

FA (folic acid); 5-FU (5-fluorouracil);

GBM (glioblastoma multiforme);

GEM (gemcitabine);

GNRs (graphene nanoribbons);

GO (graphene oxide);

GQDs (graphene quantum dots);

GR (graphene);

EPR (enhanced permeability and retention);

HA (hyaluronic acid);

HAMitP (mitochondrial ion-targeting peptide-grafted hyaluronic acid);

hDPSCs (human dental pulp stem cells);

HIF (hypoxia-inducible factor);

IBU (ibuprofen);

MDR (multidrug-resistant);

MIC (minimum inhibitory concentration);

MitP (mitochondrial ion-targeting peptide);

MRI (magnetic resonance imaging);

mTOR (mammalian target of rapamycin);

MTX (methotrexate);

MUC-1 (mucin-1);

MWCNTs (multi-walled nanotubes);

MWCNT–COOH (carboxylated multi-walled carbon nanotubes);

MTH1 (MutT homolog 1);

N-GQDs (nitrogen doped graphene quantum dots);

nanoDDSs (drug delivery nanosystems);

NIR (near infrared);

NP (nanoparticle);

NPC (nasopharyngeal carcinoma);

O-GNRs (oxidized graphene nanoribbons);

OH–GQDs (hydroxylated GQDs);

PCL (polycaprolactone);

PEG (polyethylene glycol);

PEI (polyethylenimine);

PLA (polylactic acid);

Pgp (P-glycoprotein);

PTX (paclitaxel);

PVP (poly(*N*-vinyl-2-pyrrolidone));

QDs (quantum dots);

RGD (arginine-glycine-aspartic acid);

rGO (reduced graphene oxide);

ROS (reactive oxygen species);

SWCNTs (single-walled nanotubes);

ZnPc (zinc phthalocyanine);

ZOL (zoledronic acid).
